# Supplementary material for: Conditional privatization of a public siderophore enables Pseudomonas aeruginosa to resist cheater invasion
Source: Nat Commun. 2018 Apr 11;9:1383. doi: 10.1038/s41467-018-03791-y (PMC5895777; doi:10.1038/s41467-018-03791-y)
Supplement: Supplementary file 1 — Supplementary Information [file 41467_2018_3791_MOESM1_ESM.pdf]

SUPPLEMENTARY INFORMATION

Conditional privatization of a public siderophore enables *Pseudomonas aeruginosa* to resist to cheater invasion

Jin *et al*.

Supplementary Table 1. Strains and plasmids used in this study.

	**Characteristics**	**Source**
**Strains**		
PAO1	wild type	J.D.Shrout[1]
PAO1 $-\Delta pvdA$	$pvdA$::Tet^r derivative of PAO1, Tet^r	This study
PAO1 $-\Delta pvdA \Delta fpvA$	$fpvA$::Gm^r derivative of PAO1 $-\Delta pvdA$, Gm^r Tet^r	This study
PAO1 $-\Delta pvdRT - opmQ$	$pvdRT - opmQ$::Gm^r derivative of PAO1, Gm^r	This study
PAO1-sfGFP	insertion of a single copy sfGFP driven by PA1O4O3 promoter at mini-Tn7 target site, Gm^r	This study
PAO1-mCherry	insertion of a single copy mCherry driven by PA1O4O3 promoter at mini-Tn7 target site, Gm^r	This study
PAO1 $-\Delta pvdA$- sfGFP	$pvdA$::Tet^r derivative of PAO1- sfGFP	This study
**Plasmids**		
pEX18Ap	$oriT^+$ $sacB^+$ gene replacement vector with multiple-cloning site from pUC18, Ap^r	Hoang[2]
pFGM1	Gentamicin resistance FRT vector, source plasmid of Gm^r cassette, Ap^r Gm^r	Herbert P Schweizer[3]
pUC18T-mini-Tn7T	mobilizable mini-Tn7 base vector, Ap^r Gm^r	Herbert P Schweizer[3]
mCherry-pAK1900	pAK1900 containing mCherry fused to the PA1O4O3 promoter, Ap^r	Hao Chen[4]
$\Delta pvdA_{Tet}$ − PEX	$pvdA$ allelic replacement vector in pEX18Ap, Ap^r Tet^r	This study
$\Delta fpvA_{Gen}$ − PEX	$fpvA$ allelic replacement vector in pEX18Ap, Ap^r Gm^r	This study
$\Delta pvdRT - opmQ_{Gen}$ − PEX	$pvdRT - opmQ$ allelic replacement vector in pEX18Ap, Ap^r Gm^r	This study

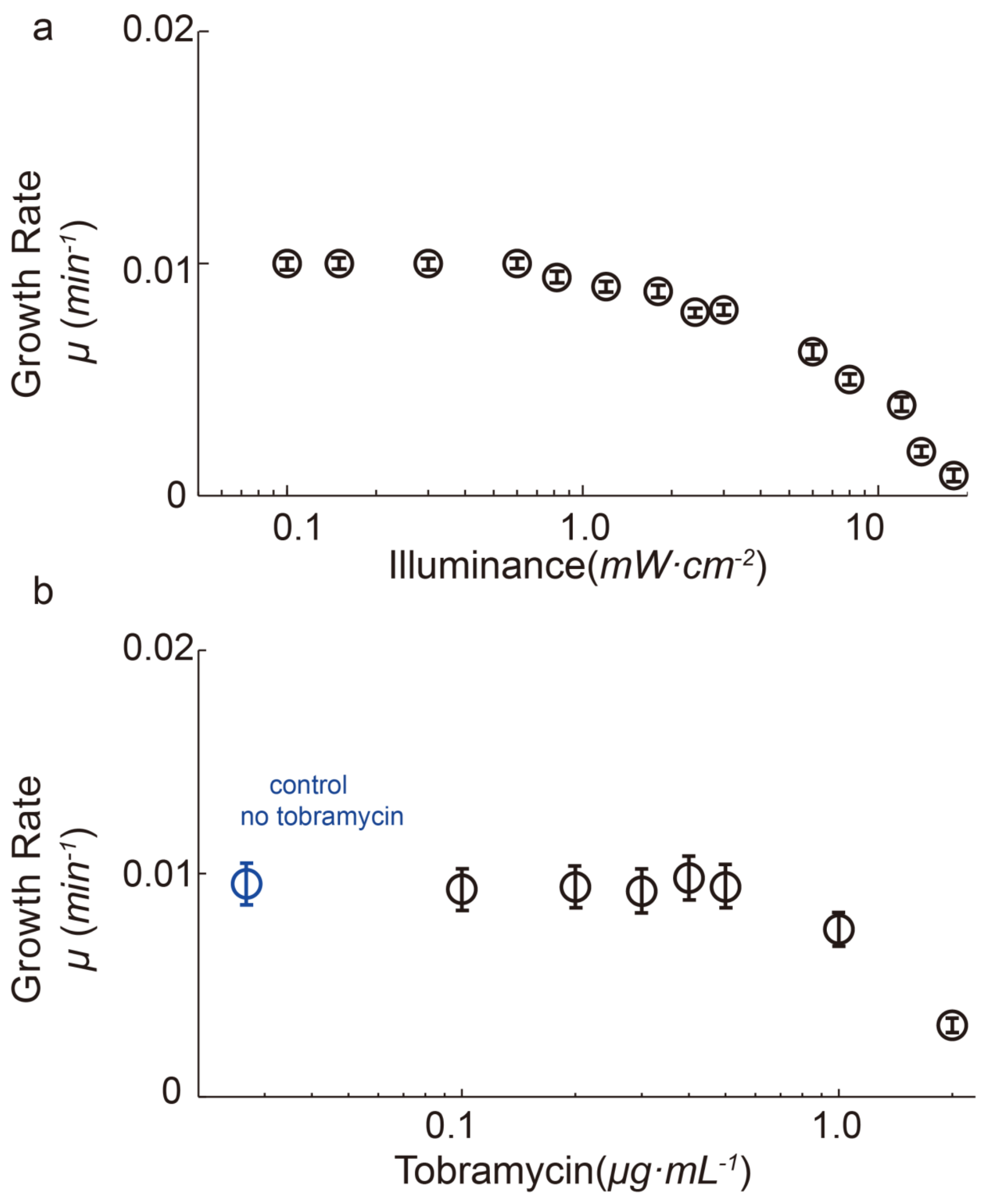

Supplementary Figure 1 | Growth rate of wide-type (PAO1) in the presence of different stress. Growth rate of PAO1 in the presence of (*a*) different photon-stresses or (*b*) antimicrobial stresses, where photon-stress or antimicrobial stress is generated by using the illumination of 405 nm laser or the treatment of tobramycin, where the black circle indicates the experiment data of average growth rate with corresponding concentration of tobramycin. Blue circle in (*b*) indicates the control group in which no tobramycin is added. The error bars in **a-b** are the standard deviation of experimental values.

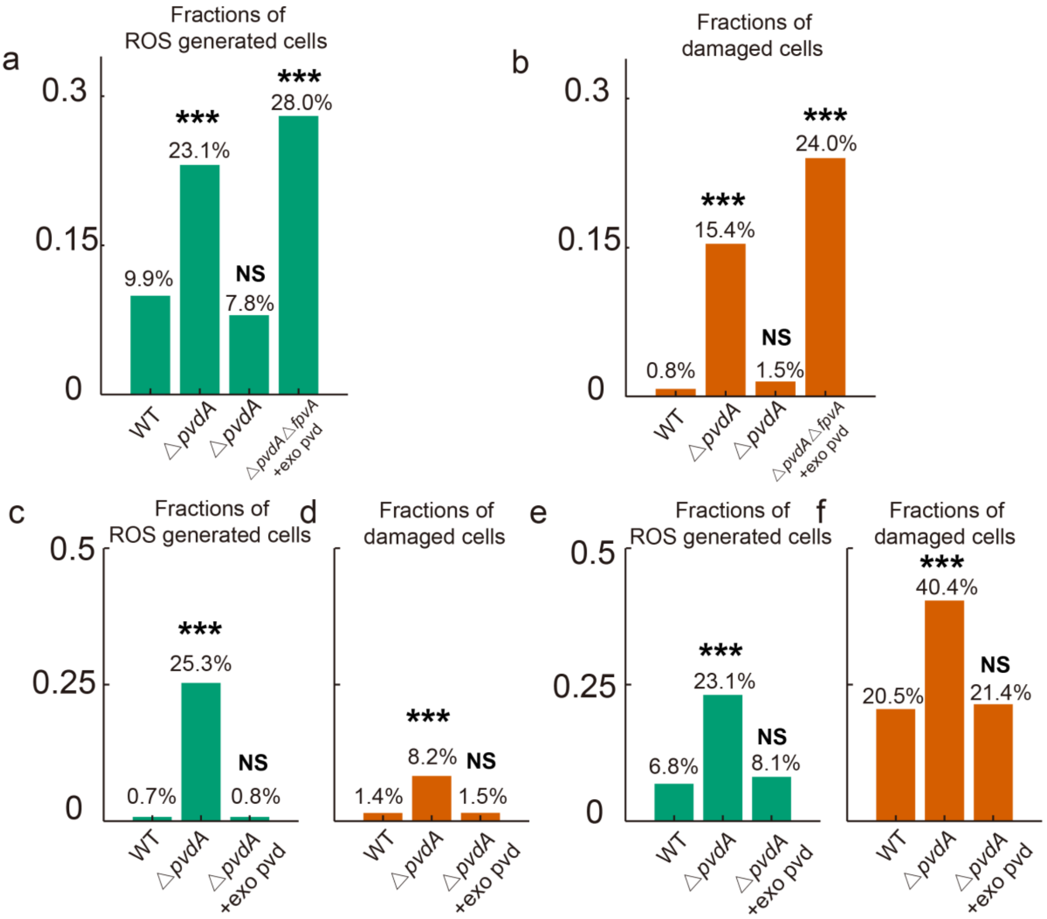

Supplementary Figure 2 | PVDI aids cell survival in the presence of antimicrobial stresses at iron-limited conditions. (a-b) Fractions of ROS generated cells (bluish green, $p = 0.1555$, $\Delta pvdA$ + exo pvd) and damaged cells (vermillion, $p = 0.9725$, $\Delta pvdA$ + exo pvd) with 1.0 $\mu g \cdot mL^{-1}$ tobramycin in iron-limited condition (SSM + $FeCl_3$ = 1.0×10^{-5} μM). **(c-d)** Fractions of ROS generated cells (bluish green, $p = 0.6375$, $\Delta pvdA$ + exo pvd) and damaged cells (vermillion, $p = 0.5276$, $\Delta pvdA$ + exo pvd) with 2.0 $\mu g \cdot mL^{-1}$ gentamycin in iron-limited condition (SSM + $FeCl_3$ = 1.0×10^{-5} μM). **(e-f)** Fractions of ROS generated cells (bluish green, $p = 0.2943$, $\Delta pvdA$ + exo pvd) and damaged cells (vermillion, $p = 0.9101$, $\Delta pvdA$ + exo pvd) with 2.0 $\mu g \cdot mL^{-1}$ gentamicin in iron-limited condition (CAA + 20 *mM* apotransferrin (fresh)). Each strain was treated for 7 hours, respectively. Treatment with 1.0 $\mu g \cdot mL^{-1}$ tobramycin led to ROS generation by a greater

fraction of $\Delta pvdA$ cells than of wild-type cells (a), and to damage in a greater fraction of $\Delta pvdA$ cells than of wild-type cells (b) in iron-limited condition (SSM + $FeCl_3$ = 1.0×10^{-5} μM). Treatment with 2.0 $\mu g \cdot mL^{-1}$ gentamicin led to ROS generation by a greater fraction of $\Delta pvdA$ cells than of wild-type cells, and to damage of a greater fraction of $\Delta pvdA$ cells than of wild-type cells in iron-limited conditions, including (c, d) SSM + $FeCl_3$ = 1.0×10^{-5} μM or (e, f) CAA + 20 mM apotransferrin (fresh). * $p < 0.05$, ** $p < 0.01$, *** $p << 10^{-6}$, NS $p > 0.1$ (one-way RM ANOVA versus wide type data on the same culture condition).

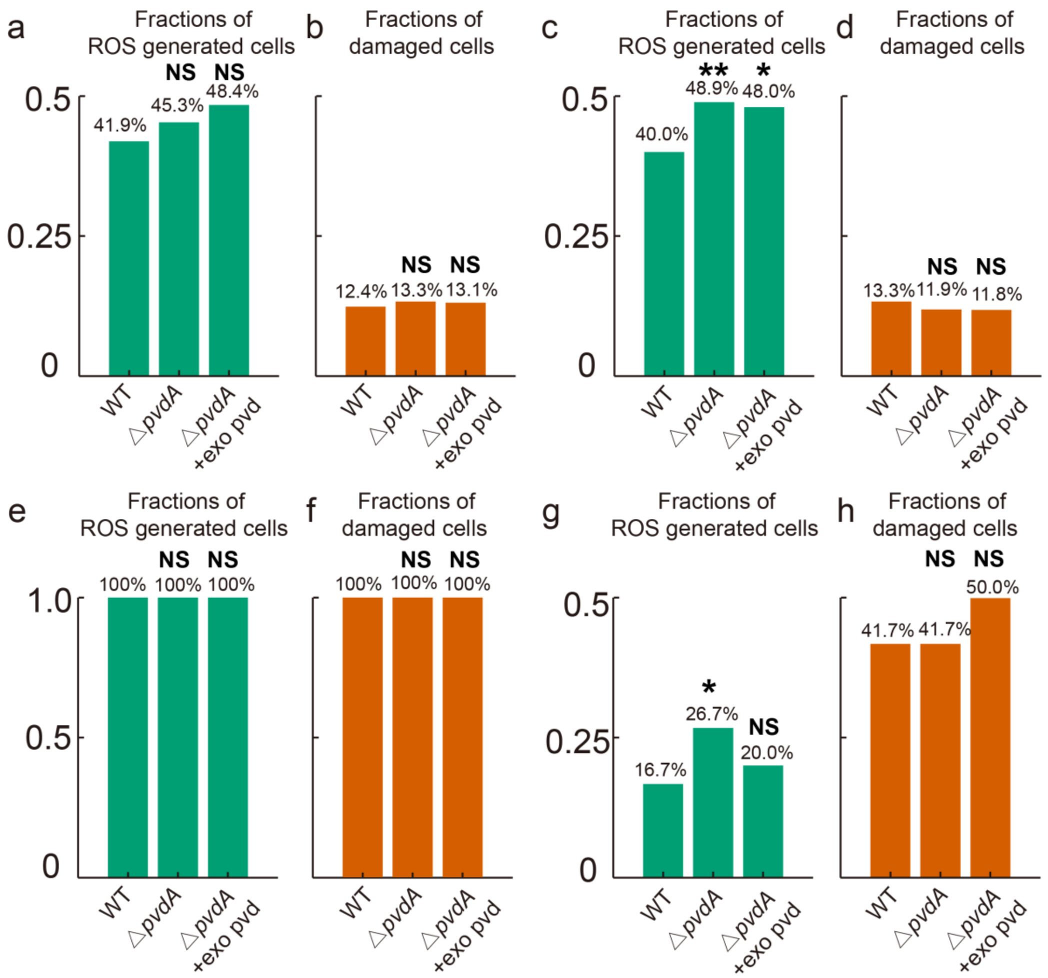

Supplementary Figure 3 | PVDI does not increase cell survival in the presence of antimicrobial stresses at iron-rich conditions. (a-b) Fractions of ROS generated cells (bluish green, p = 0.1660, $\Delta pvdA$, p = 0.1246, $\Delta pvdA$ + exo pvd) and damaged cells (vermillion, p = 0.7968, $\Delta pvdA$, p = 0.6239, $\Delta pvdA$ + exo pvd) with 1.0 $\mu g \cdot mL^{-1}$ tobramycin under iron-rich conditions (SSM + $FeCl_3$ = 100 μM). **(c-d)** Fractions of ROS generated cells (bluish green, p = 0.0087, $\Delta pvdA$, p = 0.0410, $\Delta pvdA$ + exo pvd) and damaged cells (vermillion, p = 0.9700, $\Delta pvdA$, p = 0.8349, $\Delta pvdA$ + exo pvd) with 2.0 $\mu g \cdot mL^{-1}$ gentamicin under iron-rich conditions (SSM + $FeCl_3$ = 100 μM). **(e-f)** Fractions of ROS generated cells (bluish green, p = 0.3986, $\Delta pvdA$, p = 0.1280, $\Delta pvdA$ + exo pvd) and damaged cells (vermillion, p = 0.9196, $\Delta pvdA$, p = 0.6471, $\Delta pvdA$ + exo pvd) with

2.0 $\mu g \cdot mL^{-1}$ gentamicin under iron-rich conditions (CAA). **(g-h)** Fractions of ROS generated cells (bluish green, p = 0.0337, $\Delta pvdA$, p = 0.4389, $\Delta pvdA$ + exo pvd) and damaged cells (vermillion, p = 0.2769, $\Delta pvdA$, p = 0.2025, $\Delta pvdA$ + exo pvd) with 2.0 $\mu g \cdot mL^{-1}$ gentamicin under iron-rich conditions (CAA + 20 mM apotransferrin that had been used to culture bacterial strain for 24 h). Each strain was treated for 7 hours, respectively. The fraction of cells that (a) generated ROS or (b) were damaged was similar for both $\Delta pvdA$ and wild-type strains after treatment with 1.0 $\mu g \cdot mL^{-1}$ tobramycin under iron-rich conditions (SSM + $FeCl_3$ = 100 μM). The fraction of cells that generated ROS or were damaged was similar for the $\Delta pvdA$ and wild-type strains after treatment with 2.0 $\mu g \cdot mL^{-1}$ gentamicin under iron-rich conditions, including (c, d) (SSM + $FeCl_3$ = 100 μM), (e, f) CAA or (g, h) CAA + 20 mM apotransferrin that had been used to culture bacterial strain for 24 h. * $p < 0.05$, ** $p < 0.01$, *** $p \ll 10^{-6}$, NS $p > 0.1$ (one-way RM ANOVA versus wide type data on the same culture condition).

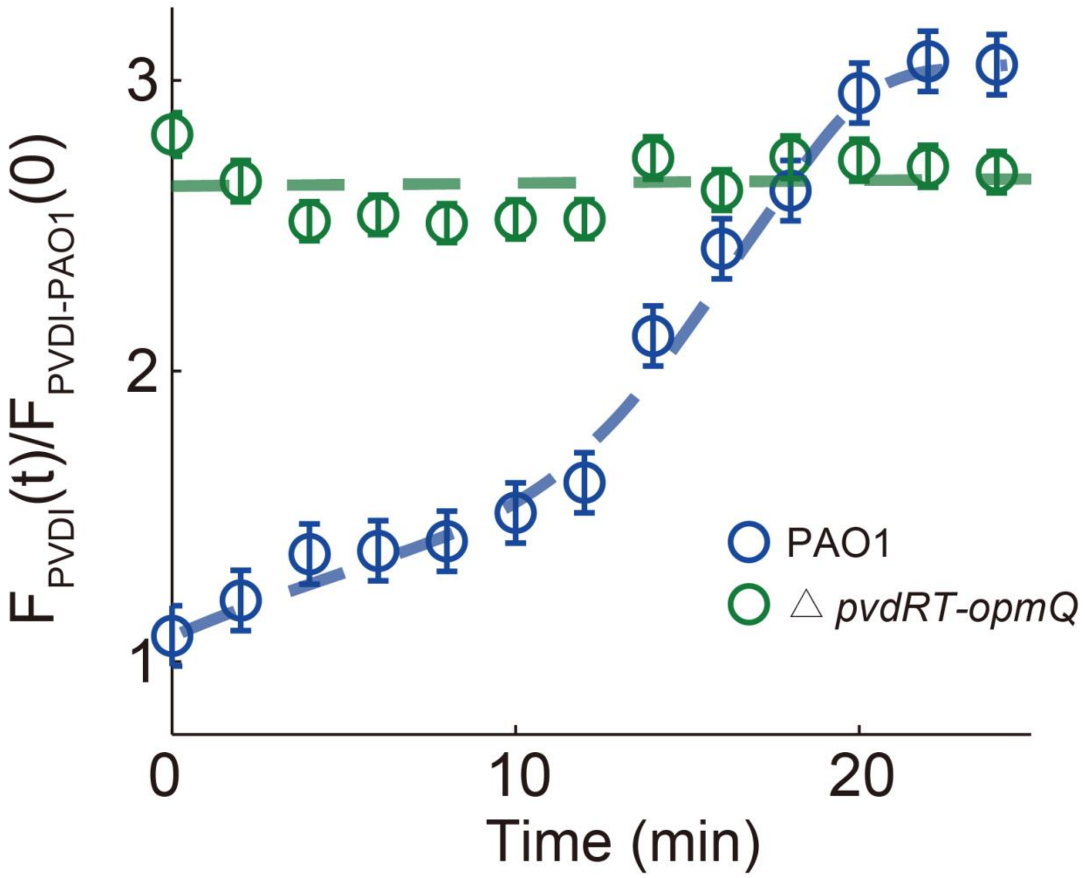

Supplementary Figure 4 |Time dependence of PVDI fluorescence arising from PAO1 or $\Delta pvdRT - opmQ$ strain under a light stimulation. Light stimulation does not result in a further accumulation of PVDI in the $\Delta pvdRT - opmQ$ strain (green), when 3.00 $mW \cdot cm^{-2}$ violet light (405 nm) was applied to illuminate cells. The error bars are the standard deviation of experimental values.

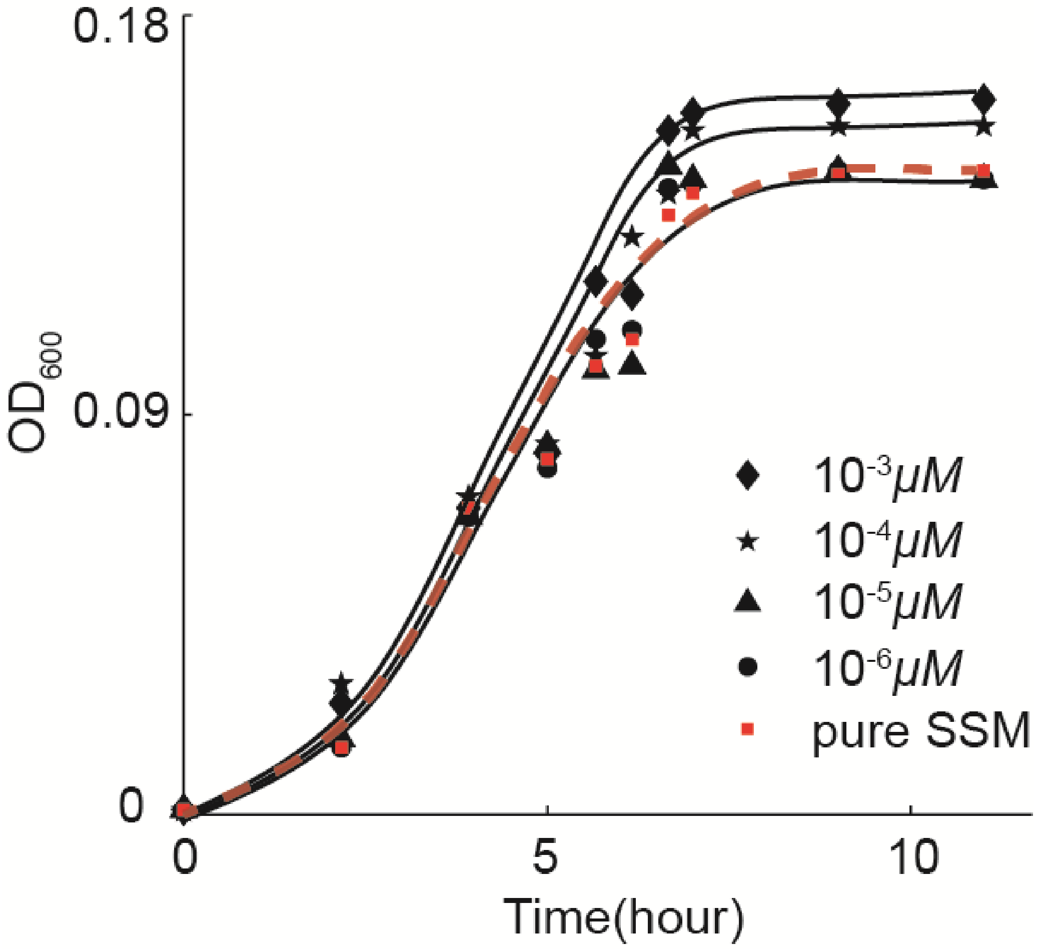

Supplementary Figure 5 | Growth curves of the wild-type strain (PAO1) in the presence of different amounts of $FeCl_3$, when the SSM medium was used. The error bars are the standard deviation of experimental values.

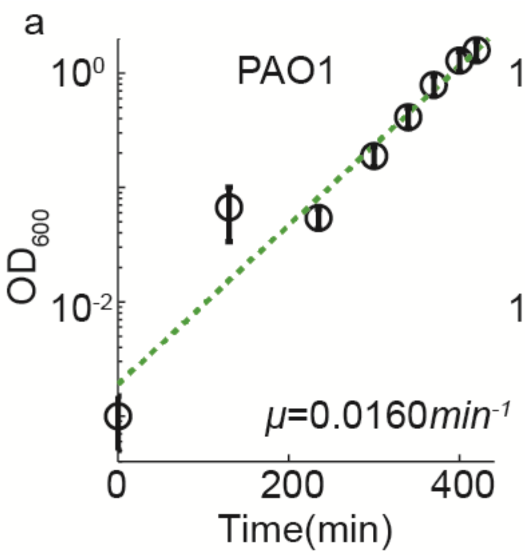

Supplementary Figure 6 | Growth curves of (a) the wild-type (PAO1) and (b) $\Delta pvdA$ strains in the presence of 1.0 μM $FeCl_3$ and 5.0 μM ePVDI at SSM medium, where the actual growth rate (μ) is determined using a linear fitting (dash line) in the semi-logarithmic plotting. The error bars are the standard deviation of experimental values.

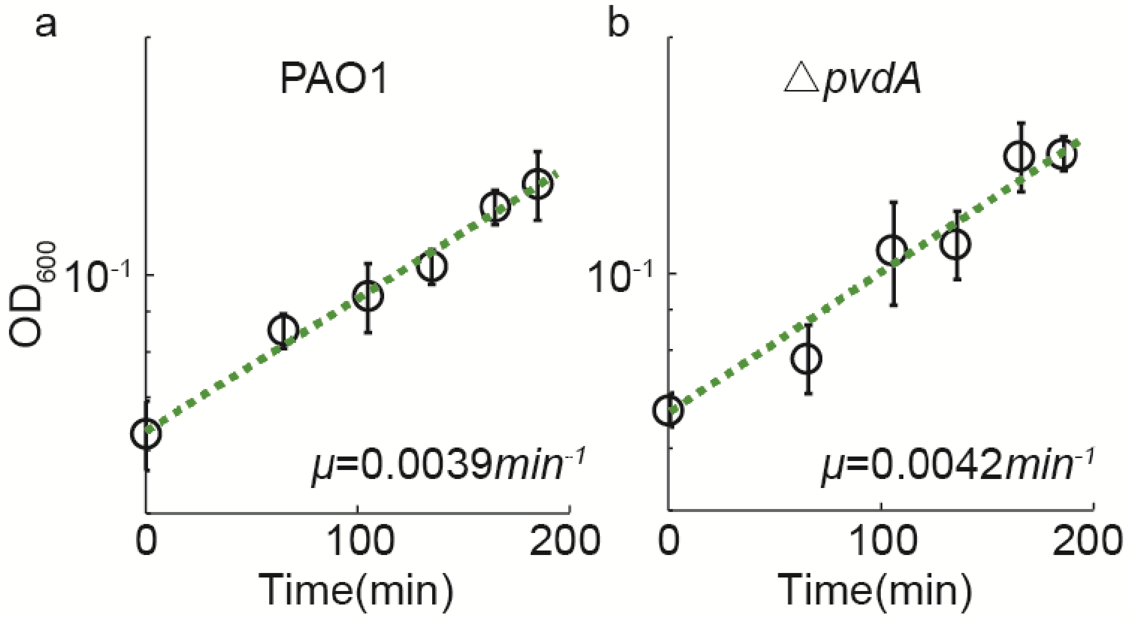

Supplementary Figure 7 | Growth curves of (a) the wild-type (PAO1) and (b) $\Delta pvdA$ strains in the presence of $1.0 \times 10^{-5}\ \mu M$ $FeCl_3$ at the SSM medium, where the actual growth rate (μ) is determined using a linear fitting (dash line) in the semi-logarithmic plotting. The error bars are the standard deviation of experimental values.

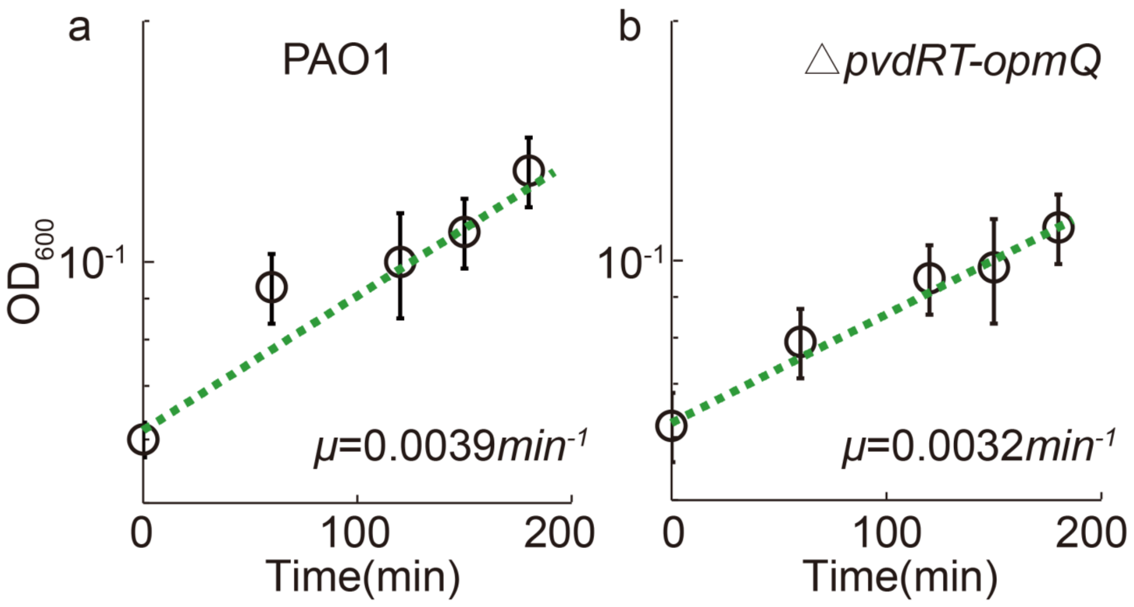

Supplementary Figure 8 | Growth curves of (a) the wild-type (PAO1) and (b) $\Delta pvdRT-opmQ$ strains in the presence of $1.0 \times 10^{-5}\,\mu M$ $FeCl_3$ at the SSM medium, where the actual growth rate (μ) is determined using a linear fitting (dash line) in the semi-logarithmic plotting. The error bars are the standard deviation of experimental values.

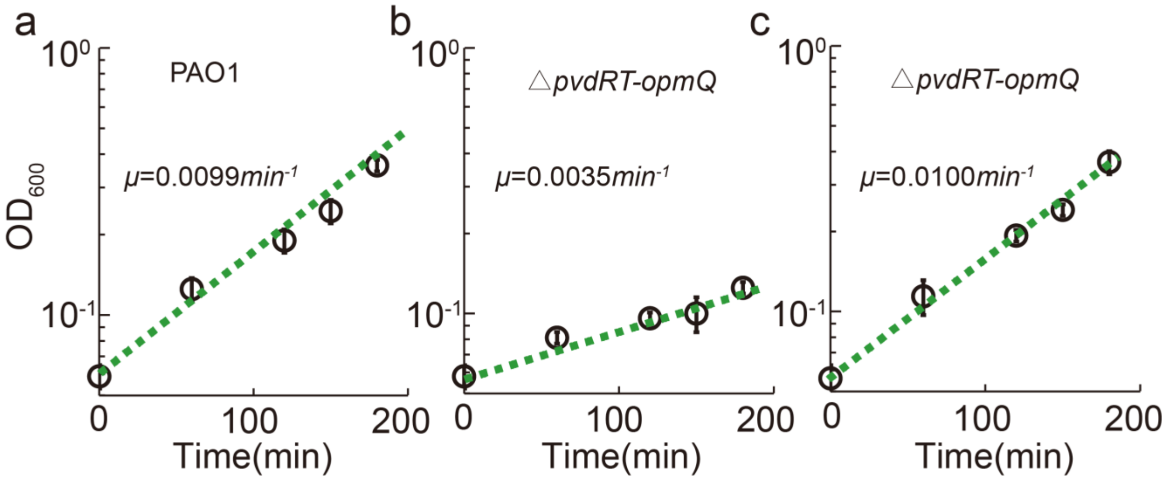

Supplementary Figure 9 | Growth curves of (a) the wild-type (PAO1) and (b) $\Delta pvdRT - opmQ$ strains in the presence of $0.1\ \mu M$ $FeCl_3$ at the SSM medium. Growth curve of (c) the $\Delta pvdRT - opmQ$ strain in the presence of $0.1\ \mu M$ $FeCl_3$ and $5.0\ \mu M$ ePVDI in SSM medium. Actual growth rate (μ) is determined using a linear fitting (dash line) in the semi-logarithmic plotting. The error bars are the standard deviation of experimental values.

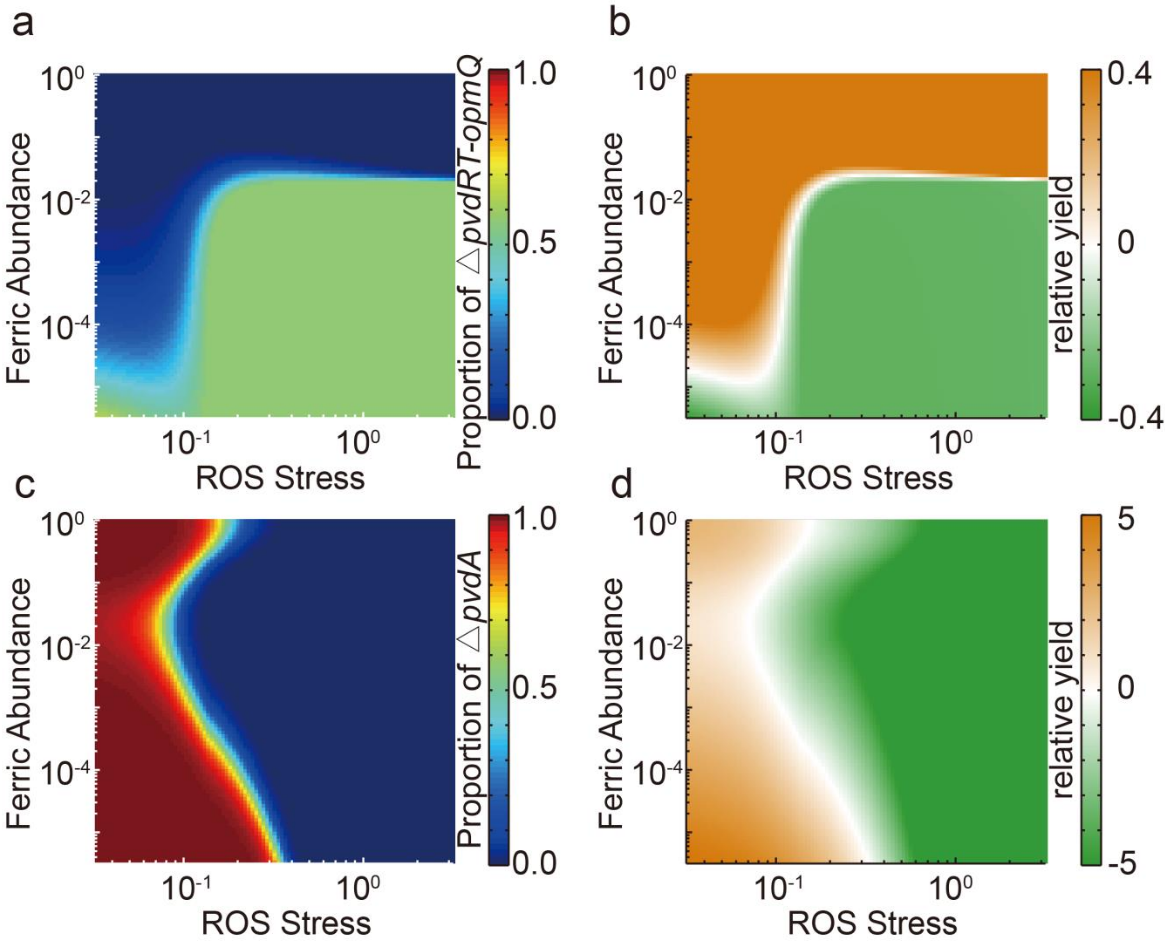

Supplementary Figure 10 | Fractions (a) or the relative yields (b) of $\Delta pvdRT - opmQ$ as a function of the ferric abundance and the normalized ROS stress in the direct competition of $\Delta pvdRT - opmQ$ and wild-type strain. Fractions (c) or the relative yields (d) of $\Delta pvdA$ as a function of the abundance of ferric and the normalized ROS stress in the direct competition of $\Delta pvdA$ and wild-type strain. Evolution time is 30 days.

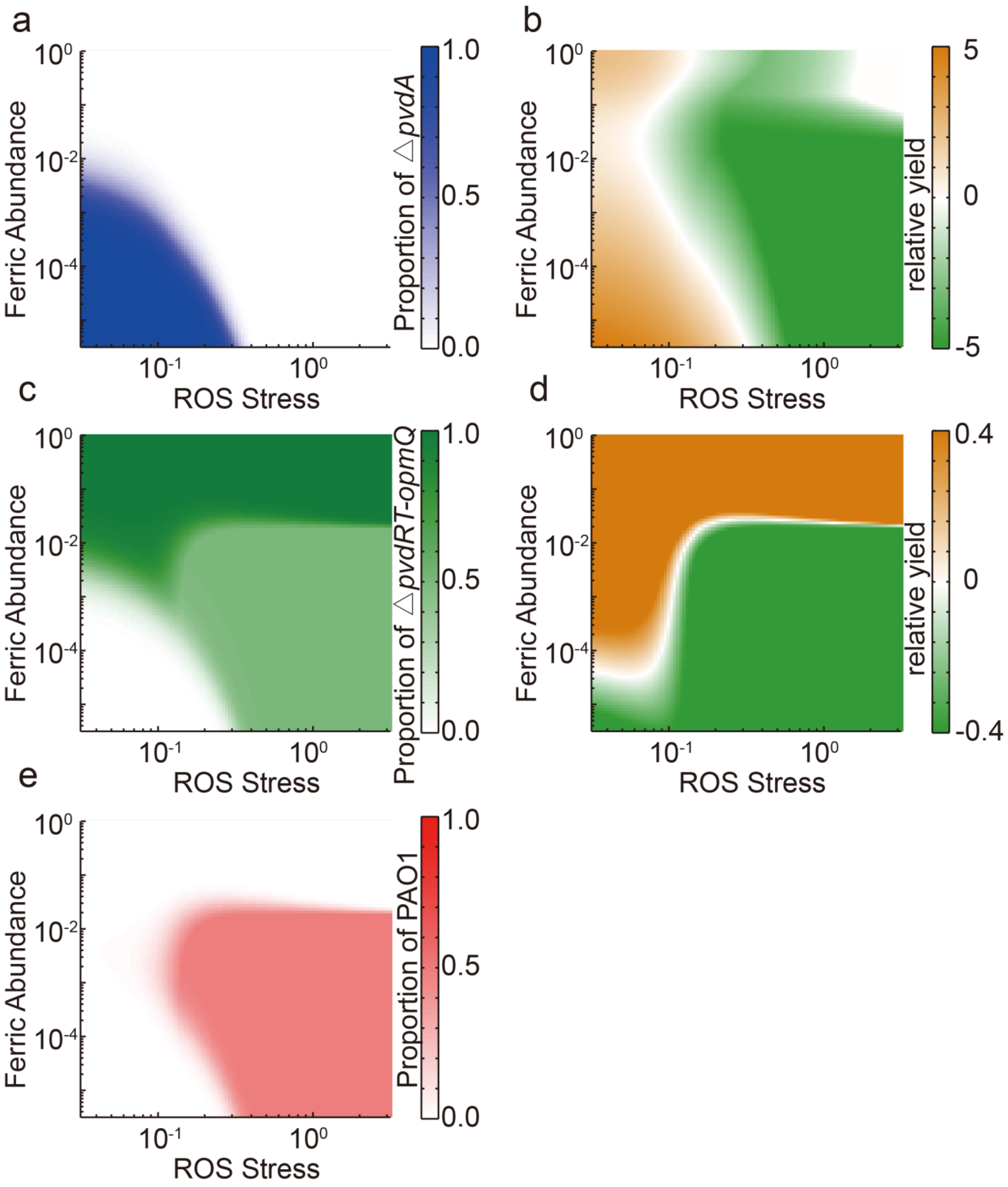

Supplementary Figure 11 | Fractions or the relative yields of $\Delta pvdA$ $(a,\ b)$, $\Delta pvdRT - opmQ$ $(c,\ d)$ or wild-type strain (e) as a function of the ferric abundance and the normalized ROS stress in the directly competition of $\Delta pvdRT - opmQ$, $\Delta pvdA$ and wild-type strain. Evolution time is 30 days.

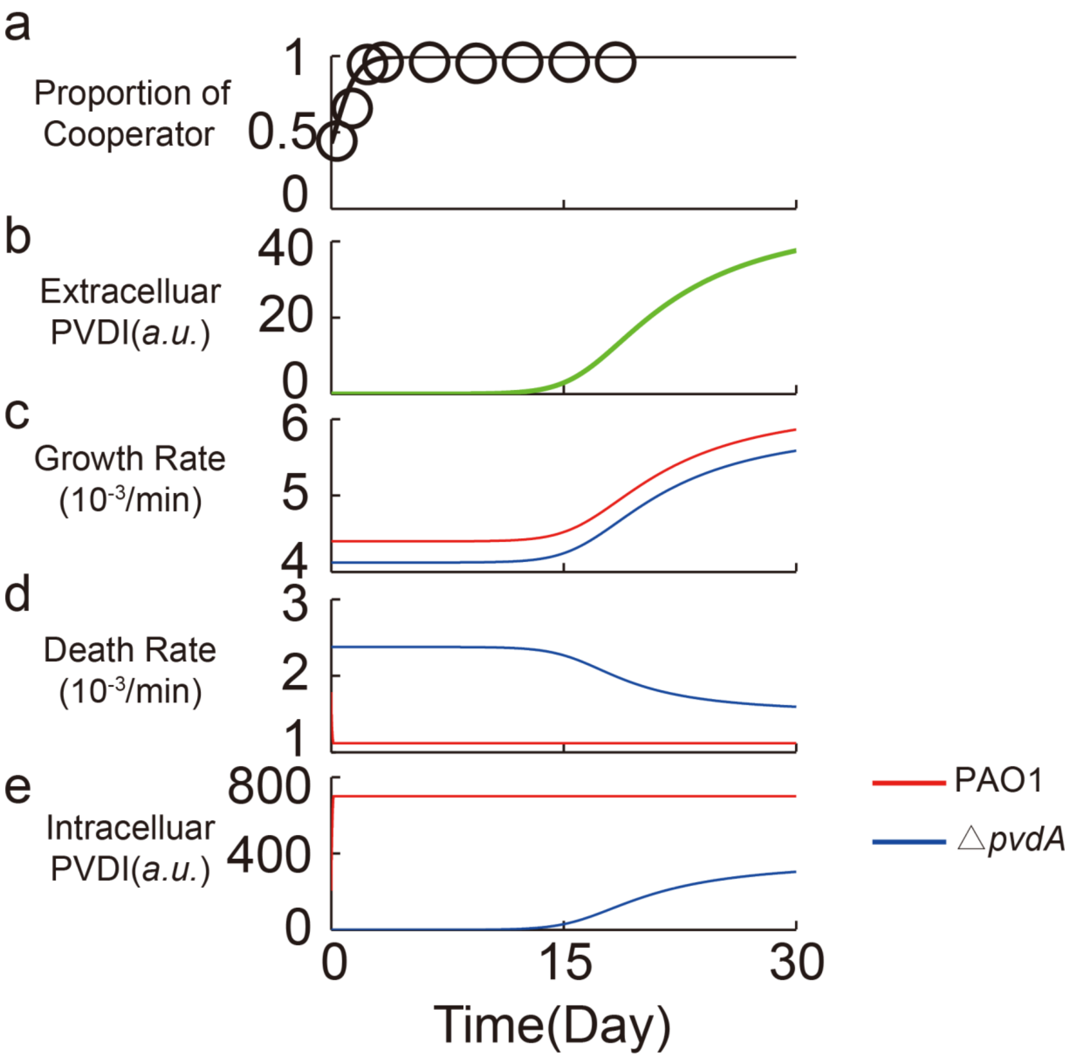

Supplementary Figure 12 | Kinetics of the fraction of the wild-type strain (a), the extracellular PVDI concentration ($[PVDI]^o$(t)) (b), the growth rate ($\mu_1(t)$ or $\mu_2(t)$) (c), the death rate ($p_1(t)$ or $p_2(t)$) (d), and the intracellular PVDI concentration ($[PVDI]_1^i$(t) or $[PVDI]_2^i$(t)) (e) of the wild-type or $\Delta pvdA$ strains during the competition at the condition of ($FeCl_3$ = $5.0 \times 10^{-3}\ \mu M$ and Tobramycin = $2.0\ \mu g \cdot mL^{-1}$), where the symbol in panel (a) indicated the results arising from the evolutionary experiments.

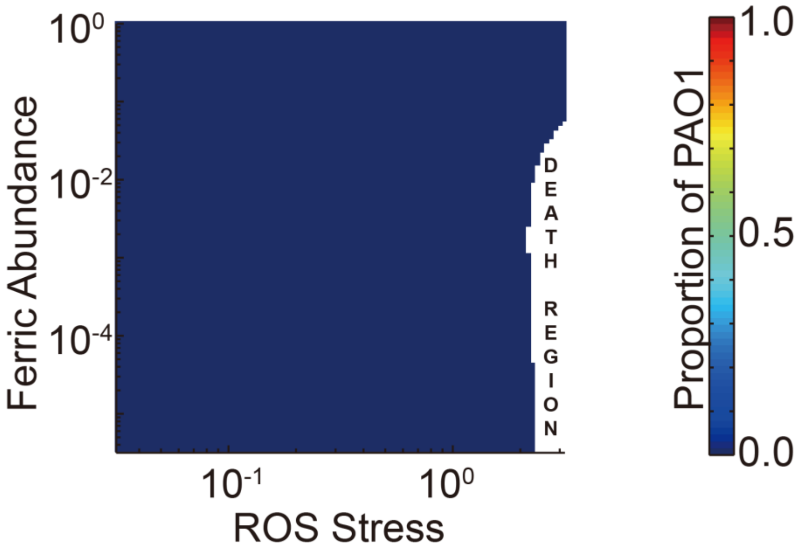

Supplementary Figure 13 | Fraction of the wild-type strain as a function of the ferric abundance and the normalized ROS stress in direct competition of the $\Delta pvdA$ and wild-type strains, when intracellular PVDI is assumed to not aid cell survival in the presence of ROS stress. The white region indicates no survival of cells.

Supplementary References

1 Shrout, J. D. *et al.* The impact of quorum sensing and swarming motility on *Pseudomonas aeruginosa* biofilm formation is nutritionally conditional. *Mol. Microbiol.* **62**, 1264-1277 (2006).

2 Hoang, T. T., Karkhoff-Schweizer, R. R., Kutchma, A. J. & Schweizer, H. P. A broad-host-range Flp-FRT recombination system for site-specific excision of chromosomally-located DNA sequences: application for isolation of unmarked *Pseudomonas aeruginosa* mutants. *Gene* **212**, 77-86 (1998).

3 Choi, K.-H. *et al.* A Tn7-based broad-range bacterial cloning and expression system. *Nat. Methods* **2**, 443 (2005).

4 Chen, H. *et al.* The *Pseudomonas aeruginosa* multidrug efflux regulator MexR uses an oxidation-sensing mechanism. *Proc. Natl. Acad. Sci. USA* **105**, 13586-13591 (2008).
