# Supplementary material for: Conditional privatization of a public siderophore enables Pseudomonas aeruginosa to resist cheater invasion
Source: Nat Commun. 2018 Apr 11;9:1383. doi: 10.1038/s41467-018-03791-y (PMC5895777; doi:10.1038/s41467-018-03791-y)
Supplement: Supplementary file 3 — Description of Additional Supplementary Files [file 41467_2018_3791_MOESM3_ESM.pdf]

Description of Additional Supplementary Files

File Name: Supplementary Movie 1
Description: Photon-stress ($6.00\ mW \cdot cm^{-2}$) quickly triggers the accumulation of pyoverdine in the periplasms of *P. aeruginosa* in 4 minutes. (Bar: 2 μm)

File Name: Supplementary Movie 2
Description: ROS was generated quickly in single *P. aeruginosa* with photon-stress ($6.00\ mW \cdot cm^{-2}$) in 8 minutes. (Bar: 2 μm)

File Name: Supplementary Movie 3
Description: Accumulation of pyoverdine in the periplasms allows more wild-type *P. aeruginosa* cells to survive in the presence of environmental stress ($3.00\ mW \cdot cm^{-2}$). (Bar: 2 μm)

File Name: Supplementary Movie 4
Description: Environmental stress ($3.00\ mW \cdot cm^{-2}$) leads more $\Delta pvdA$ cells to be damaged without the accumulation of pyoverdine. (Bar: 2 μm)

File Name: Supplementary Movie 5
Description: Exogenous addition of PVDI of allows more $\Delta pvdA$ cells to survive in presence of environmental stress ($3.00\ mW \cdot cm^{-2}$). (Bar: 2 μm)

File Name: Supplementary Movie 6
Description: Environmental stress ($3.00\ mW \cdot cm^{-2}$) leads more $\Delta pvdA\Delta fpvA$ cells to be damaged even with the exogenous addition of PVDI. (Bar: 2 μm)
